# Carnosic acid alleviates high-altitude cerebral edema by suppressing oxidative stress and microglia activation-induced neuroinflammation

**DOI:** 10.3389/fphar.2026.1892158

**Published:** 2026-09-23

**Authors:** Junlan Gao, Ningwei Che, Jian Yu

**Affiliations:** 1 Department of Critical Care Medicine, Second Hospital of Dalian Medical University, Dalian, China; 2 Department of Neurosurgery, Second Hospital of Dalian Medical University, Dalian, China

**Keywords:** carnosic acid, high-altitude cerebral edema, neuroinflammation, NF-κB–NLRP3, oxidative stress

## Abstract

**Background:**

Oxidative stress and neuroinflammation constitute key pathological contributors to high-altitude cerebral edema (HACE). Carnosic acid (CA) exerts important roles in neurodegeneration and brain injury, but its function in HACE remains elusive.

**Methods:**

High-altitude hypobaric hypoxic (HH)-mimicked HACE rats were administered carnosic acid. The effects on hematological parameter changes, blood–brain barrier (BBB) permeability, energetic metabolism disorder, cognitive and motor impairment, oxidative stress, and microglia-mediated neuroinflammation were explored. The function of carnosic acid in microglia-mediated damage to astrocytes and endothelial cells under hypoxia was investigated.

**Results:**

Carnosic acid dose-dependently normalized aberrant hematological parameter changes, brain water content, and histopathological changes in HH-mimicked HACE rats. Moreover, HH conditions enhanced BBB permeability and BBB-related aquaporin 4 (AQP4) expression and decreased BBB-related occludin and zonula occludens-1 (ZO-1) expression, which were reversed after carnosic acid. Carnosic acid alleviated energetic metabolism disorder and improved cognitive and motor impairment in HACE rats. Carnosic acid attenuated HH-evoked oxidative stress by inhibiting reactive oxygen species (ROS) and malondialdehyde levels and increased antioxidant glutathione and superoxide dismutase levels. Carnosic acid suppressed HH-induced microglia activation and pro-inflammatory response. Additionally, aberrant activation of the NF-κB–NLRP3 inflammasome axis in HACE rats was also inhibited by carnosic acid. Carnosic acid inhibited hypoxia-induced pro-inflammatory microglia activation by the NF-κB–NLRP3 pathway. Moreover, carnosic acid also attenuated hypoxia-activated microglia-induced AQP4 expression in astrocytes and damage to the endothelial tight junction.

**Conclusion:**

Carnosic acid may attenuate the pathological progression of HACE by restraining oxidative stress and microglia activation-induced neuroinflammation via the NF-κB–NLRP3 axis, supporting it as a promising neuroprotective intervention for HACE.

## 1 Introduction

With the increasing prevalence of travel and work, more than 100 million people have been reported to ascend to high-altitude regions worldwide annually (Garrido et al., 2021). Acute mountain sickness (AMS) affects approximately 10%–51% of people when they are exposed to altitudes above 2,500 m in either acute or prolonged circumstances (Derstine et al., 2023; Gatterer et al., 2024). High-altitude cerebral edema (HACE) is an extreme form/end-stage manifestation of AMS at altitudes above 4,000 m and can affect 0.5%–2% of individuals (Turner et al., 2021). Although HACE is an infrequent form of AMS, it represents a potentially life-threatening encephalopathy for tourists and plateau practitioners due to its rapid progression and severe clinical symptoms, including weakness, consciousness impairment, headache, and loss of coordination (Derstine et al., 2023; Turner et al., 2021). Despite abundant research about HACE, the current therapeutic interventions for repairing injury caused by HACE remain a great challenge in clinical practice. Therefore, more effective therapeutic strategies against HACE are urgently needed.

Hypobaric hypoxia (HH) is widely accepted as the characterization of rapid exposure to high altitude and constitutes the initial culprit in evoking cerebral vascular leakage and cell swelling that promotes the progression of HACE. Currently, oxidative stress and neuroinflammation are recognized as the key pathological mechanisms for the initiation and development of HACE (Pena et al., 2022; Shushanyan et al., 2024). The preceding study has confirmed that stimulation with HH will facilitate reactive oxygen species (ROS) accumulation and evoke oxidative stress leading to brain injury and cellular edema, worsening the pathological progression of cerebral edema (He et al., 2024; Shushanyan et al., 2024). Accumulating evidence has corroborated neuroinflammation as the pathophysiological cause of HACE. Intriguingly, HH of altitude may induce a pro-inflammatory response by activating the microglia, the major immune cells in the central nervous system (Song et al., 2016; Wang et al., 2022). A previous study revealed that the systemic inflammatory response can accelerate the onset of hypoxic cerebral edema by interacting with microglial-evoked inflammatory signaling and aggravate the brain edema associated with HACE by inducing blood–brain barrier (BBB) disruption (Song et al., 2016; Zhou et al., 2017).

Carnosic acid is a naturally abundant phenolic diterpene derived from rosemary (*Rosmarinus officinalis*) that is traditionally used for the treatment of various diseases, such as headaches and inflammatory diseases. Numerous preclinical experiments substantiate that carnosic acid exerts remarkable and diverse bioactive properties, including anti-inflammatory and anti-tumor activity. For instance, carnosic acid can protect against lipopolysaccharide-induced hepatic and intestinal inflammation in poultry (Wan et al., 2024). Carnosic acid has a gastroprotective effect in indomethacin-induced gastric ulceration in rats by suppressing oxidative stress and inflammatory response (Danisman et al., 2023). Moreover, carnosic acid can effectively attenuate inflammatory response and oxidative stress in experimental arthritis (Chrastina et al., 2022). Notably, emerging evidence has revealed neuroprotection of carnosic acid in several neurodegenerative disorders. For example, carnosic acid protects against 6-hydroxydopamine-induced neurotoxicity in Parkinson’s disease by exerting its anti-apoptotic and anti-oxidative efficacy (Iorio et al., 2022; Lin et al., 2020). Additionally, nanocarrier-packaged carnosic acid can alleviate glia-mediated neuroinflammation and improve cognitive function in an Alzheimer’s disease model (Yi-Bin et al., 2022). However, its effects on HACE remain unclear. In this study, we constructed a HACE animal model and analyzed the efficacy and potential molecular mechanism of carnosic acid in the HACE model.

## 2 Experimental procedures

### 2.1 Experimental animal and ethics statement

Male Sprague-Dawley (SD) rats (weight of 180–220 g) were selected and purchased from the Center of Laboratory Animals, Dalian Medical University. All rats were housed for 1 week of adaptive feeding in standard conditions (constant temperature of 23 °C ± 2 °C, relative humidity of 55%–60%, and 12:12 h light to dark cycles) prior to experiments. All experimental animals were allowed food and water *ad libitum.* All procedures and protocols during animal experiments were overseen by the National Institutes of Health (NIH) Guide for the Care and Use of Laboratory Animals and were ethically approved by the Institutional Animal Care and Use Committee of Dalian Medical University (No. AEE23010). All reasonable efforts were carried out to minimize animal suffering.

### 2.2 HACE animal model and administration

Rats were randomly divided into the following groups (n = 6 in each group): a sham group, HACE groups, and HACE and CA groups. To simulate the HACE environment, rats were exposed to a high-altitude hypobaric hypoxia condition as previously described (Jing et al., 2020). Briefly, rats were placed in an experimental low-pressure, low-oxygen environment system (Shanghai Yuyan Scientific Instruments Co. Ltd., LP-1500; Shanghai, China) to simulate a 6,000 m altitude (9.9 kPa, equal to 9.8% O_2_) for 3 days. The simulated altitude was reached at a rate of 10 m/min. In the sham group, rats were maintained in normal laboratory conditions with standard chow and water. For the HACE and CA groups, before exposure to the HACE environment, rats were given various intragastric doses of CA (20 mg/kg, 40 mg/kg, and 80 mg/kg, once a day) for 2 days, and then were administered CA once a day for 3 days under HACE conditions.

### 2.3 Detection of hematological parameters

To assess the efficacy of CA in hematological parameters under HACE conditions, the blood specimens were collected in ethylene diamine tetraacetic acid (EDTA) tubes. Then, the hematological parameters were determined using a XN-550™ Automated Hematology Analyzer (Sysmex, Japan), including white blood cells, red blood cells, hemoglobin, hematocrit, lymphocytes, granulocytes, monocytes, platelets, and mean cell volume.

### 2.4 Evaluation of brain water content

To collect the left hemispheres, rats were anesthetized and sacrificed by cervical dislocation without suffering. Samples were immediately weighed to obtain the wet weight using a precision electronic balance. Subsequently, the specimens were dried to prepare the constant dry weights at 60 °C for 72 h. The percentage of BWC was calculated using the following formula: BWC (%) = (wet weight − dry weight)/wet weight × 100%.

### 2.5 Assay of blood–brain barrier permeability

To evaluate effects on BBB permeability, rats were anesthetized and injected intravenously with 8 mL/kg Evans blue solution (2%; Sigma, MO, United States) via the tail. Approximately 1 h later, rats were deeply anesthetized and perfused with PBS Tokyo, Japan (pH 7.4) to remove the intravascular Evans blue dye until the drainage was colorless. After sacrifice, brain tissue was removed and incubated with dimethylformamide at 50 °C. Forty-eight hours later, the samples were centrifuged at 1,500 × g for 5 min to prepare supernatants, followed by the determination of absorbance of Evans blue at 630 nm using a spectrophotometer (Thermo Fisher Scientific, Waltham, MA, United States).

### 2.6 Histopathological analysis

Brain tissue specimens from various groups were collected, and all samples were fixed in 4% paraformaldehyde. Subsequently, specimens were dehydrated with graded ethanol and embedded in paraffin, followed by cutting into 5 μm-thick sections. Hematoxylin and eosin (H&E) staining was then performed to analyze the histopathological changes under light microscopy.

### 2.7 Assessment of energy metabolism

To determine the energy metabolism changes in the rat model, the brain tissues were collected and homogenized. Then, the specimens were centrifuged to prepare supernatants. The levels of adenosine triphosphate (ATP), pyruvate kinase (PK), and ATPase in the supernatants were measured using commercial kits (Nanjing Jiancheng Bioengineering Institute, Nanjing, China). All procedures were performed according to the manufacturer’s instructions.

### 2.8 Detection of oxidative stress

The levels of reactive oxygen species (ROS) and malondialdehyde (MDA) in tissue specimens were determined to assess the oxidative stress levels. To detect the levels of ROS, the prepared tissue supernatants were incubated with 10 µM of cell-permeative probe 2′, 7′-dichlorodihydrofluorescein diacetate (DCFH-DA; Beyotime, Shanghai, China) to yield fluorescent DCF. Approximately 0.5 h later, the excitation wavelength (490 nm) and emission wavelength (525 nm) were captured to detect the fluorescence intensity using a fluorometric microplate reader to assess ROS levels. For MDA detection, specimens were treated with 0.2 mL of MDA detection solution (Beyotime) at 100 °C. Then, the OD532 nm was determined to evaluate the MDA level.

### 2.9 Assay of anti-oxidative stress

To assess anti-oxidative stress status, the levels of superoxide dismutase (SOD) and glutathione (GSH) were determined. For the SOD assay, all protocols were performed according to the instructions of the commercial SOD Detection Kit (Nanjing Jiancheng Bioengineering Institute). The absorbance at 450 nm was measured to analyze SOD levels. For the GSH assay, the prepared tissue supernatants were mixed with the reaction reagents (Nanjing Jiancheng Bioengineering Institute) and centrifuged for 10 min. Then, the absorbance at 405 nm was detected using a microplate reader.

### 2.10 Maze test for cognitive assessment

To assess the effects of carnosic acid on cognitive function in the HACE model, a T-maze test was conducted as previously reported (Kauser et al., 2013). In brief, rats were trained for 1 week to habituate to a T-maze experiment. Once rats had become familiar with the apparatus and food reward, they underwent additional training to ensure stable performance with achievement of a criterion of more than 80% correct choices in two consecutive sessions, with a 24-h interval between each session. Then, the successfully trained rats were randomly divided into the sham group, a HACE group, and HACE/CA groups. Rats were tested after various treatments. The cognitive function was assessed by calculating the % of correct choices.

### 2.11 Rotarod test

The rotarod test was carried out to evaluate the locomotory coordination of rats as previously described (Gong et al., 2018). Before testing, all rats were allowed to familiarize themselves with the rotarod training until they could maintain balance. Following the indicated treatments, rats were acclimated to the rotarod at a uniform speed of 4 rpm/min for 5 min. They were then tested with the gradually increasing speed from 4 rpm/min to 50 rpm/min over a period of 5 min, with a maximum duration of 10 min for each trial. The trial came to an end when the rat fell off the apparatus.

### 2.12 Immunofluorescence

For the immunofluorescence assay, brain tissue sections were treated with 0.5% Triton X-100 for 15 min. Then, the section samples were incubated with 5% bovine serum albumin solution at room temperature for 0.5 h to block non-specific binding. Subsequently, the primary antibodies against rat IBA-1 (Abcam Inc., Cambridge, MA, United States) were added for further incubation at 4 °C overnight. After rinsing three times, the Alexa Fluor-conjugated anti-rat second antibodies were added at room temperature for 1 h. 4′,6-Diamidino-2-phenylindole (DAPI) was used to stain the cellular nucleus. The specimens were then analyzed using a fluorescence microscope (Nikon, Tokyo, Japan), and the fluorescence intensity was examined using ImageJ software.

### 2.13 Microglia and astrocyte culture and treatment

Rat primary microglia and astrocytes were purchased from Procell Biotechnology (Wuhan, China). All cells were maintained in Dulbecco’s modified Eagle medium (DMEM) (Gibco, Carlsbad, CA, United States) medium supplemented with 10% FBS. Cells between passages 1 and 3 were used for subsequent experiments. For culture, microglia were treated with various doses of carnosic acid (1–100 μM), pyrrolidinedithiocarbamate (PDTC) (NF-κB inhibitor, 50 μmol/L), or 1 μg/mL lipopolysaccharide (LPS) (an NF-κB activator) under hypoxia exposure (1% O_2_) for 48 h. Then, the conditioned medium (CM) from microglia under various treatments for 48 h was collected. The astrocytes were then treated with CM from microglia under hypoxia conditions for 24 h.

### 2.14 Transepithelial electrical resistance and transcytosis analysis

To evaluate the integrity of the tight junction of microvascular endothelial cells, the transepithelial electrical resistance (TEER) and transcytosis analysis were performed as previously reported (Wang et al., 2022). Briefly, rat cerebral microvascular endothelial cells were obtained from BNCC (Henan, China). For the TEER assay, endothelial cells were seeded on 24-transwell inserts, and microglia were placed on the top of the endothelium. After co-culture at 1% O_2_ for 24 h, cells were treated with Hanks’ Balanced Salt Solution (HBSS) for 0.5 h. Then, the Millicell ERS-2 Epithelial Volt-ohm Meter (Merck Millipore) was used to measure the total resistance of co-cultured cells. Resistance of HBSS was defined as black resistance. The TEER was analyzed as previously reported (Wang et al., 2022); lower resistance indicated injury to the tight junctions of cells. To perform the transcytosis analysis, 250 μg/mL Dextran-40 was added to the inserts. One hour later, a spectrophotometer was used to determine the concentration of dextran in the collected medium in the lower plate.

### 2.15 Quantitative reverse transcription-polymerase chain reaction

After treatment with carnosic acid under hypoxic conditions, the mRNA from microglia was prepared using TRIzol reagent (Invitrogen, Carlsbad, CA, United States), followed by the synthesis of cDNA. Then, real-time PCR was carried out to detect the mRNA levels of IBA-1 and iNOS. All procedures were performed according to the instructions of a SYBR Premix Ex Taq™ II Kit (TaKaRa, Dalian, China) on an ABI PRISM 7000 sequence detection system (Applied Biosystems, Foster City, CA, United States). The specific primers were used as follows: IBA-1 (sense, 5ʹ-CTC​CTC​CAA​GAC​CCA​AAC​TAA​C-3ʹ; anti-sense, 5ʹ-CGT​AGG​GAA​GAG​AGA​GTC​TGA​A-3ʹ) and iNOS (sense, 5ʹ-GGG​TTT​GGA​GAG​ACA​GAA​GTG-3ʹ; anti-sense, 5ʹ-GGG​AGA​GGA​GAG​AGA​TTC​AGT​AG-3ʹ). GAPDH served as an internal control. The relative mRNA expression was calculated using the 2^−ΔΔCt^ method.

### 2.16 Determination of inflammatory cytokine levels by ELISA assay

The levels of the inflammatory cytokines TNF-α, IL-6, IL-10, and IL-1β were detected in brain tissue and microglial supernatants. All procedures were carried out in accordance with the recommendations of the commercial Rat ELISA Detection Kits (Beyotime).

### 2.17 Western blotting

The prepared brain tissue, astrocyte, and endothelial cell samples were lysed to extract total protein using the RIPA lysis buffer (Beyotime). Then, an equal amount of protein (30 µg in each lane) was denatured and separated by 12% SDS-PAGE, followed by transferring to a PVDF membrane. The membranes were then blocked and incubated with primary antibodies against rat occludin (1:1,000), ZO-1 (1:1,000), AQP4 (1:1,000), IBA-1 (1:2000), NLRP3 (1:1,000), ASC (1:1,500), p-p65 NF-κB (1:1,000), and p65 NF-κB (1:10,000) at 4 °C overnight. All antibodies were purchased from Abcam. Then, the horseradish peroxidase (HRP)-conjugated secondary antibodies were used for further incubation for 2 h. Finally, enhanced chemiluminescence (ECL) (Beyotime) was applied to visualize the binding protein bands. Immunoreactive band intensities were quantified using ImageJ software.

### 2.18 Statistical analysis

All experimental data were repeated at least three times and analyzed using GraphPad Prism 10.1.0. All results were represented as mean ± SD. Statistical significance was set as *p* < 0.05. The statistical comparisons for three or more groups were carried out using ANOVA with Tukey *post hoc* tests.

## 3 Results

### 3.1 CA attenuates HACE-induced hematological changes

There were obvious increases in white blood cells (Figure 1A), red blood cells (Figure 1B), hemoglobin (Figure 1C), and hematocrit (Figure 1D) in the hypobaric hypoxia (HH)-mimicked HACE rat model relative to the sham group. Concomitantly, the percentages of lymphocytes (Figure 1E), granulocytes (Figure 1F), and monocytes (Figure 1G) in blood samples were also upregulated when rats were exposed to the HH environment. HH conditions also elevated platelets (Figure 1H) and mean cell volume (Figure 1I). Notably, CA dose-dependently ameliorated HH-induced elevation of hematological parameters; however, no obvious difference was noted between the 40 mg/kg and the 80 mg/kg-treated groups (Figures 1A–I). Therefore, the dose of 40 mg/kg CA was chosen for the next experiments.

**FIGURE 1:**
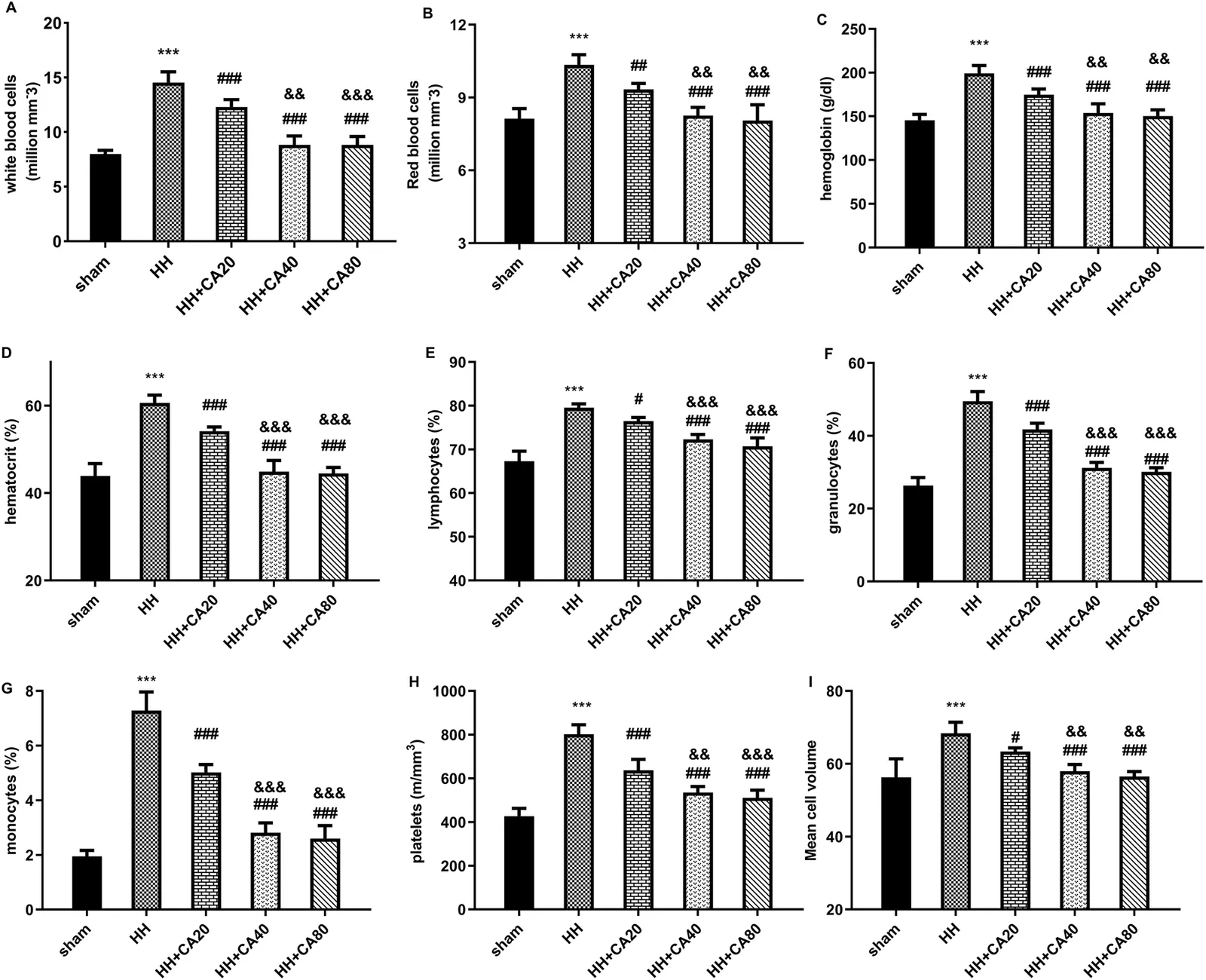
Carnosic acid dose-dependently attenuates hematological changes in HACE rats. HACE rats were constructed by exposure to an HH environment and treated with various doses of carnosic acid (20 mg/kg, 40 mg/kg, and 80 mg/kg). Three days later, the hematological parameters of white blood cells **(A)**, red blood cells **(B)**, hemoglobin **(C)**, hematocrit **(D)**, percentages of lymphocytes **(E)**, granulocytes **(F)**, and monocytes **(G)**, platelets **(H)**, and mean cell volume **(I)** were measured. ^***^ *p* < 0.001 vs. sham groups. ^#^ *p* < 0.05 and ^###^ *p* < 0.001 vs. HACE groups. ^&&^ *p* < 0.05 and ^&&&^ *p* < 0.001 vs. HACE and CA (20 mg/kg) groups.

### 3.2 Treatment with CA alleviates brain edema and BBB injury in rats exposed to a HACE environment

We further determined brain water content and revealed that HH exposure elevated brain water content relative to the sham group, which was attenuated by CA (Figure 2A). HE staining revealed normal arrangement and structure of neurons and cerebral capillaries in brain tissue from the sham group; however, HACE rats exhibited histopathological changes of dilated perivascular space and edema (Figure 2B). Moreover, Evans blue (EB) staining assay confirmed the elevation of EB in HH groups, indicating that HH exposure increased permeability of the BBB (Figure 2C). Administration of CA significantly ameliorated BBB permeability in HACE rats. Concomitantly, the protein expression of occludin and ZO-1 was decreased (Figures 2D–F), and BBB permeability protein AQP4 was increased in HH groups (Figures 2D,G), which were abrogated by CA (Figures 2D–G).

**FIGURE 2:**
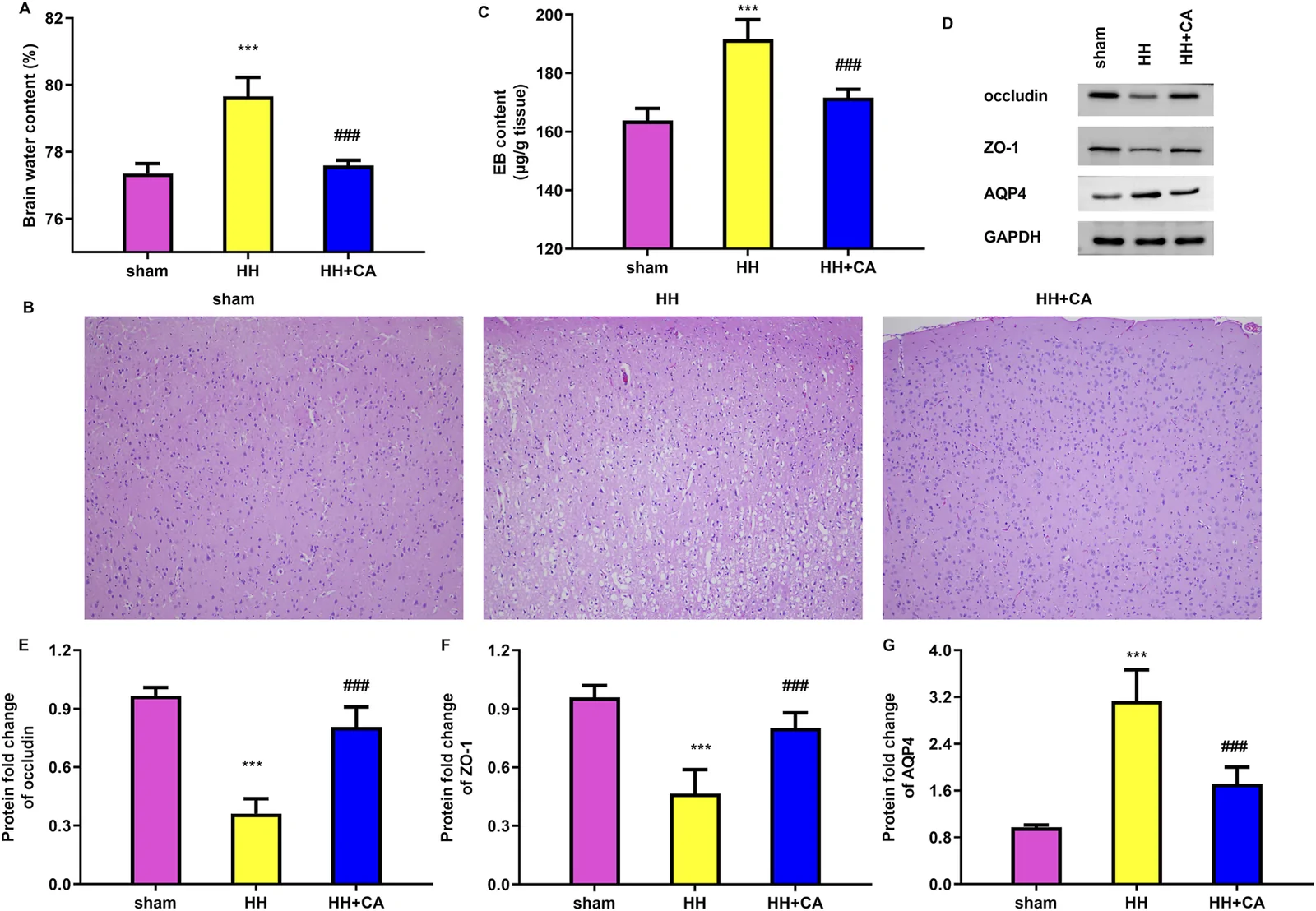
Administration of carnosic acid ameliorates brain edema in HACE rats. HACE rats received 40 mg/kg carnosic acid. **(A)** Brain water content was then determined. **(B)** HE staining was performed to observe the histopathological changes. **(C)** Evans blue (EB) level was analyzed. **(D–G)** Protein expression of occludin, ZO-1, and AQP4 was determined by Western blotting assay. ^***^ *p* < 0.001 vs. sham groups. ^###^ *p* < 0.001 vs. HACE groups.

### 3.3 CA administration improves energetic metabolism disorder and cognitive and motor impairment in HACE rats

Further assays substantiated that rats exposed to HH conditions had decreased ATP (Figure 3A), Na^+^-K^+^-ATPase (Figure 3B), and Ca^2+^-Mg^2+^-ATPase activities (Figure 3C) in brain tissues relative to the sham group. Simultaneously, increased levels of PK were determined in HH rat groups (Figure 3D). CA treatment greatly ameliorated HH-evoked decreases of ATP, Na^+^-K^+^-ATPase, and Ca^2+^-Mg^2+^-ATPase, and increases of PK, indicating the protective efficacy of CA in alleviating energetic metabolism disorder. The maze test revealed that HACE rats had a lower percentage of correction relative to the sham groups, indicating the cognitive decline of HACE rats; however, this decline was effectively improved by CA administration (Figure 3E). The rotarod test confirmed that the time at which the mice fell off the rolling rod was reduced in HH groups relative to the sham group, which was reversed by CA (Figure 3F).

**FIGURE 3:**
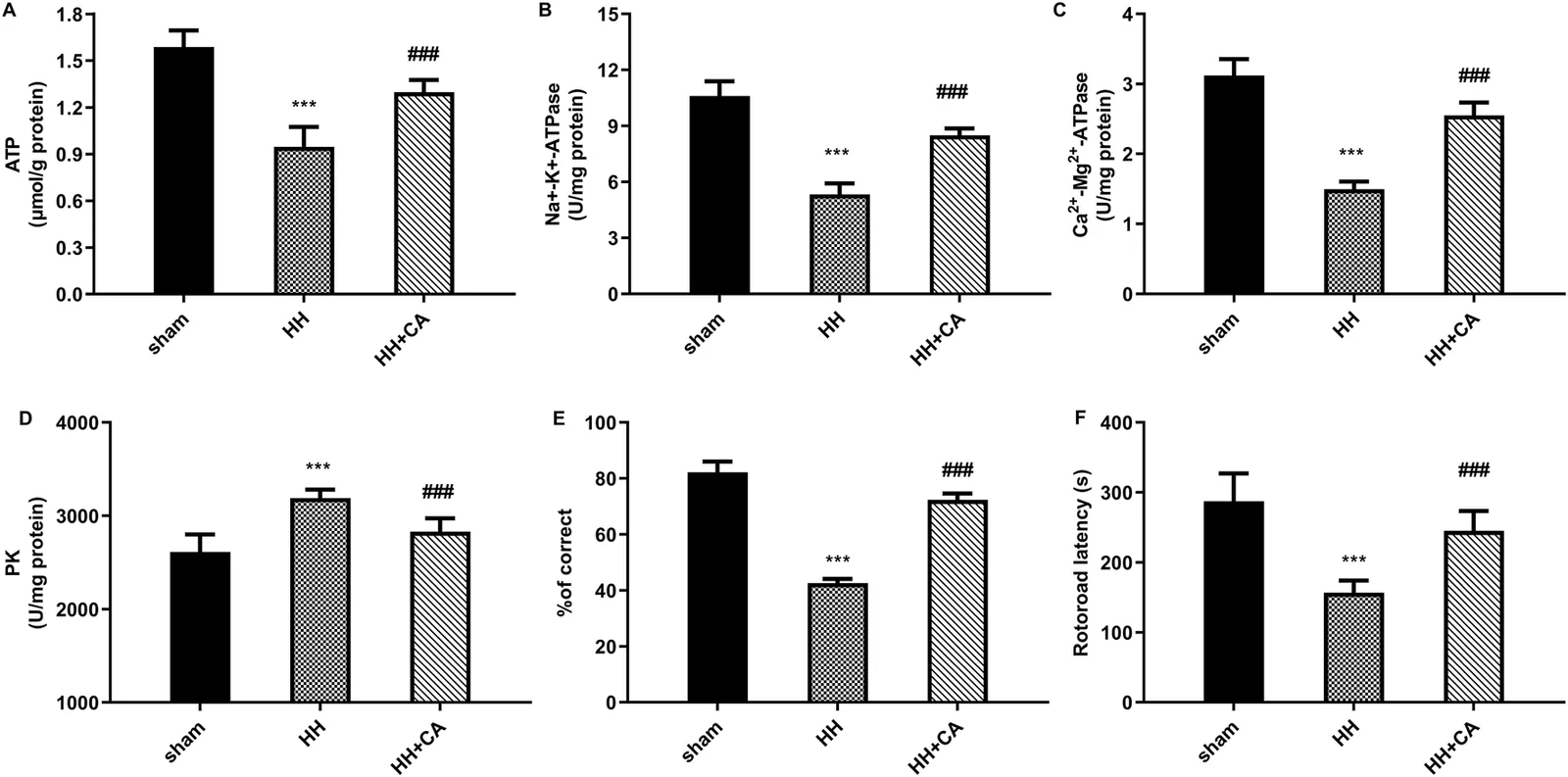
Carnosic acid treatment improves HACE-evoked energetic metabolism disorder, cognitive, and motor impairment. **(A)** ATP levels. **(B)**Na^+^-K^+^-ATPase activity. **(C)** Ca^2+^-Mg^2+^-ATPase activity. **(D)** PK levels. **(E)** Maze test and rotarod test **(F)** were carried out to evaluate the effects of carnosic acid on cognitive and motor function in HACE rats, respectively. ^***^ *p* < 0.001 vs. sham groups. ^###^ *p* < 0.001 vs. HACE groups.

### 3.4 Administration of CA attenuates oxidative stress in the HACE model

As shown in Figures 4A,B, compared with the sham group, the levels of oxidative stress markers ROS (Figure 4A) and MDA (Figure 4B) were dramatically upregulated in rats with HACE, revealing the high oxidative stress levels in HACE rats. Rats exposed to HH conditions had lower SOD activity (Figure 4C) and GSH levels (Figure 4D) than those in the sham groups, indicating the lower anti-oxidative stress status in HACE rats. Administration of CA significantly attenuated HH-increased ROS and MDA levels and enhanced anti-oxidative stress SOD and GSH levels in HACE rats.

**FIGURE 4:**
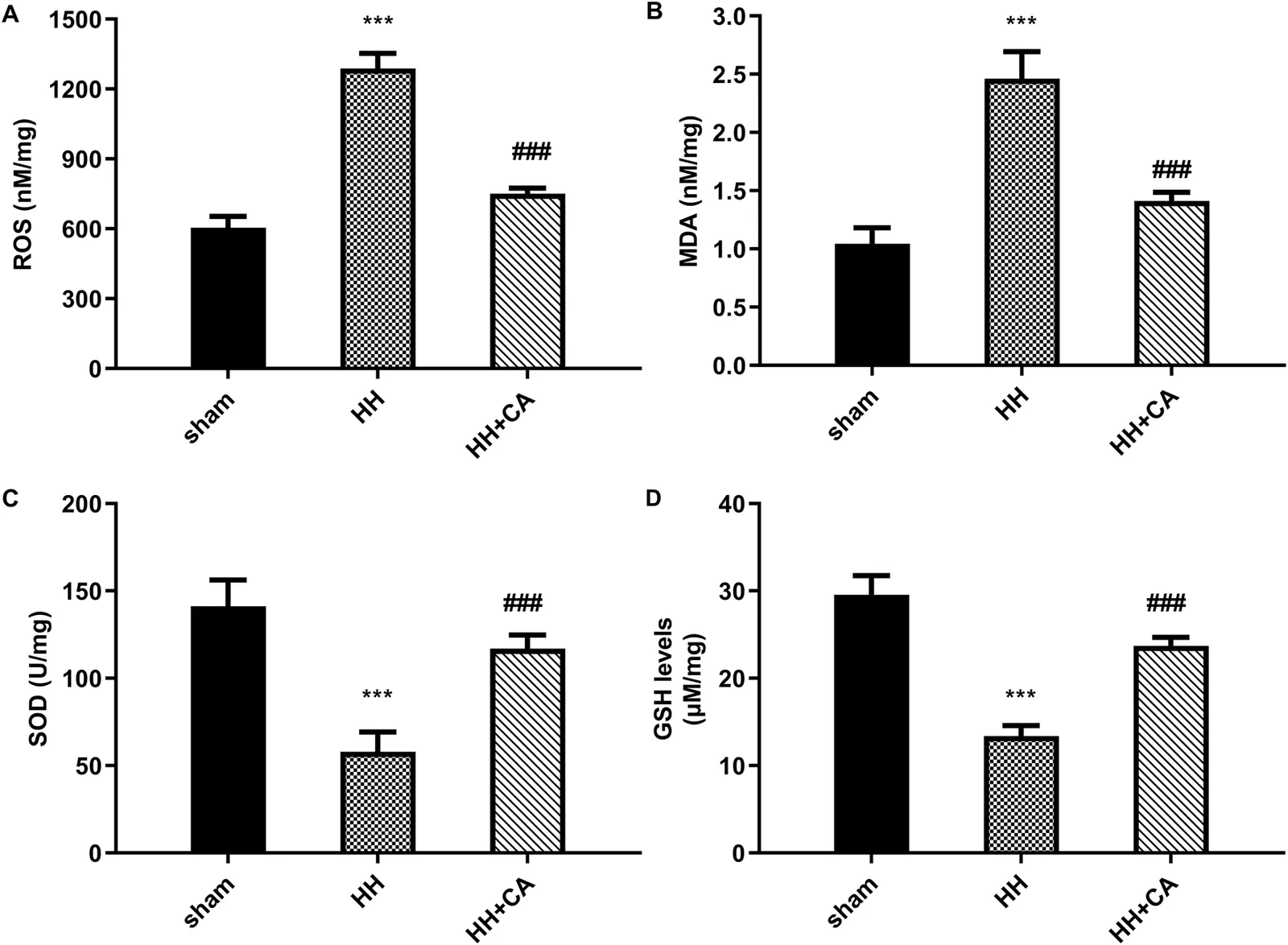
Carnosic acid suppresses oxidative stress evoked by HACE conditions. Following treatment with carnosic acid, the levels of ROS **(A)**, MDA **(B)**, SOD **(C)**, and GSH **(D)** were determined in HACE rats. ^***^ *p* < 0.001 vs. sham groups. ^###^ *p* < 0.001 vs. HACE groups.

### 3.5 CA restrains microglial activation and neuroinflammation in the HACE model

Convincing evidence reveals the key roles of neuroinflammation in the pathological progression of HACE. We analyzed the effects of CA on microglial activation in HACE rats. As shown in Figure 5A, the increased numbers of microglial marker IBA-1^+^ cells were reversed by CA. CA reduced HH-elevated protein expression of IBA-1 (Figure 5B). Compared with the sham groups, rats with HACE had increased levels of pro-inflammatory cytokine TNF-α (Figure 5C), IL-6 (Figure 5D), and IL-1β (Figure 5E), concomitant with the decreased levels of anti-inflammatory IL-10 (Figure 5F). CA treatment antagonized HH-increased pro-inflammatory cytokine levels and HH-reduced IL-10 levels.

**FIGURE 5:**
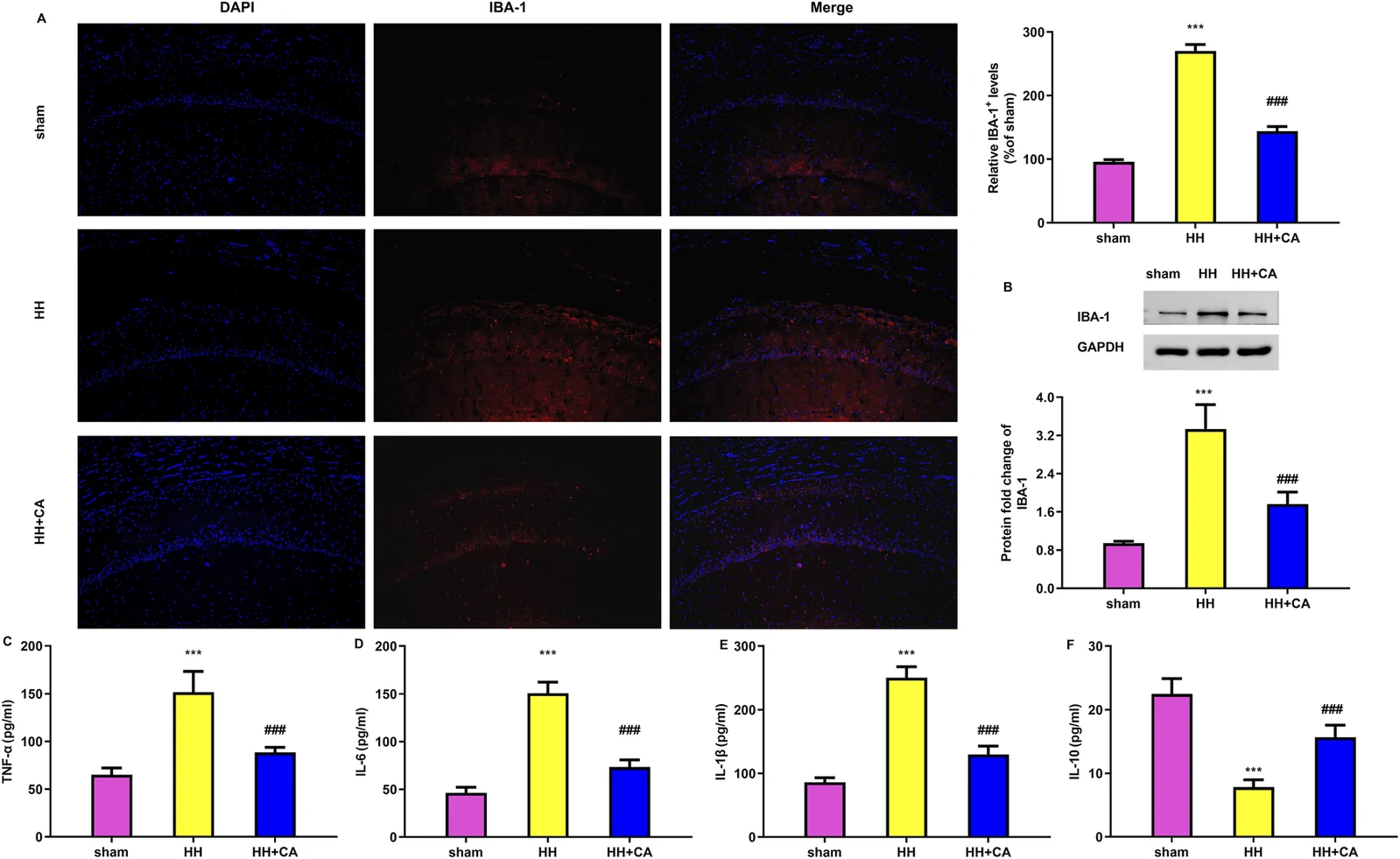
Administration of carnosic acid antagonizes HACE-induced microglia activation and neuroinflammation in a rat model. **(A,B)** Immunofluorescence staining **(A)** and Western blotting **(B)** were performed to determine the expression of microglial marker IBA-1. **(C–F)** An ELISA assay was conducted to determine the levels of TNF-α **(C)**, IL-6 **(D)**, IL-1β **(E)**, and IL-10 **(F)**. ^***^ *p* < 0.001 vs. sham groups. ^###^ *p* < 0.001 vs. HACE groups.

### 3.6 Treatment with CA inhibits the activation of the NF-κB–NLRP3 inflammasome signaling in HACE rats

Accumulating research confirms the critical function of the NF-κB signaling in the development of HACE. Thus, we analyzed the activation of this pathway in HACE rats. Compared with the sham group, exposure to HH enhanced the protein expression of p-p65 NF-κB (Figures 6A,B), which was reversed by CA treatment. Moreover, the downstream protein expression of NLRP3 (Figures 6A,C) and ASC (Figures 6A,D) was increased in rats with HACE relative to the sham group; these increases were reversed by CA. Therefore, these data reveal that CA may attenuate HACE-induced activation of the NF-κB-NLRP3 inflammasome pathway.

**FIGURE 6:**
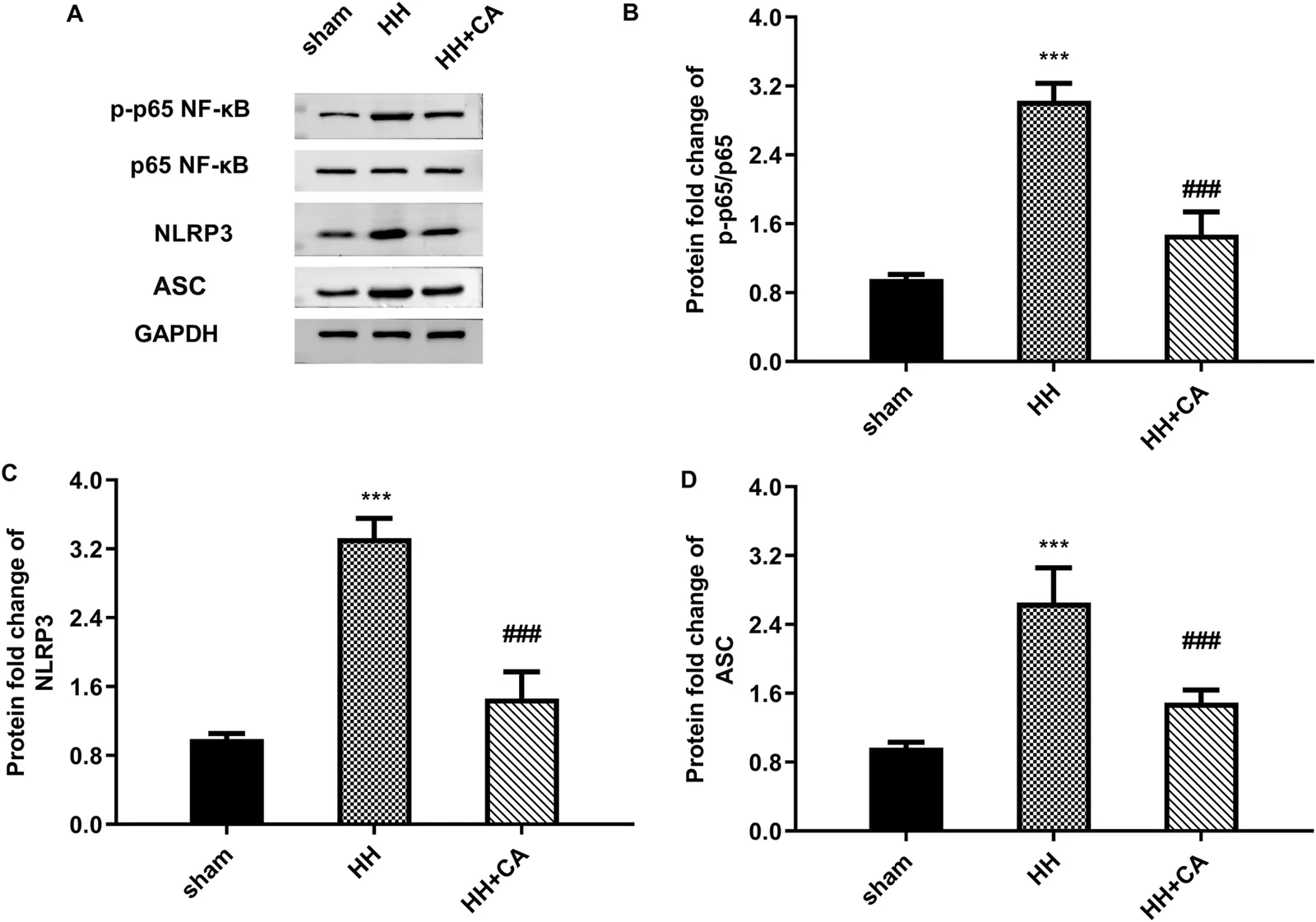
Treatment with carnosic acid reduces the activation of NF-κB-NLRP3 inflammasome signaling in HACE rats. **(A–D)** The protein expression of p-p65 NF-κB, p65 NF-κB, NLRP3, and ASC was analyzed in carnosic acid-treated HACE rats. ^***^ *p* < 0.001 vs. sham groups. ^###^ *p* < 0.001 vs. HACE groups.

### 3.7 CA inhibits pro-inflammatory microglia activation under hypoxia exposure by inhibiting the NF-κB signaling

Further in vitro assays confirmed that hypoxia obviously increased the transcription of the marker IBA-1, which was dose-dependently inhibited by CA; no dramatic difference was observed between the 20 µM and 50 μM CA groups (Figure 7A). The dose of 20 μM was selected for the following experiments. Simultaneously, the enhanced protein expression of IBA-1 under hypoxic conditions was also suppressed by CA (Figure 7B). The mRNA levels of proinflammatory M1 microglial marker iNOS were increased after hypoxia exposure, which was abrogated by CA (Figure 7C). The elevated levels of pro-inflammatory TNF-α (Figure 7D) and IL-6 (Figure 7E) in microglia under hypoxia conditions were reversed by CA. Hypoxia enhanced activation of the NF-κB-NLRP3 inflammasome, which was inhibited by CA (Figure 7F). Notably, inhibiting the NF-κB pathway by PDTC also restrained hypoxia-induced increases of pro-inflammatory microglia (Figure 7G), TNF-α (Figure 7H), and IL-6 (Figure 7I). Reactivating this signaling by LPS offset the inhibitory effects of CA on hypoxia-increased iNOS expression, TNF-α, and IL-6 levels (Figures 7G–I).

**FIGURE 7:**
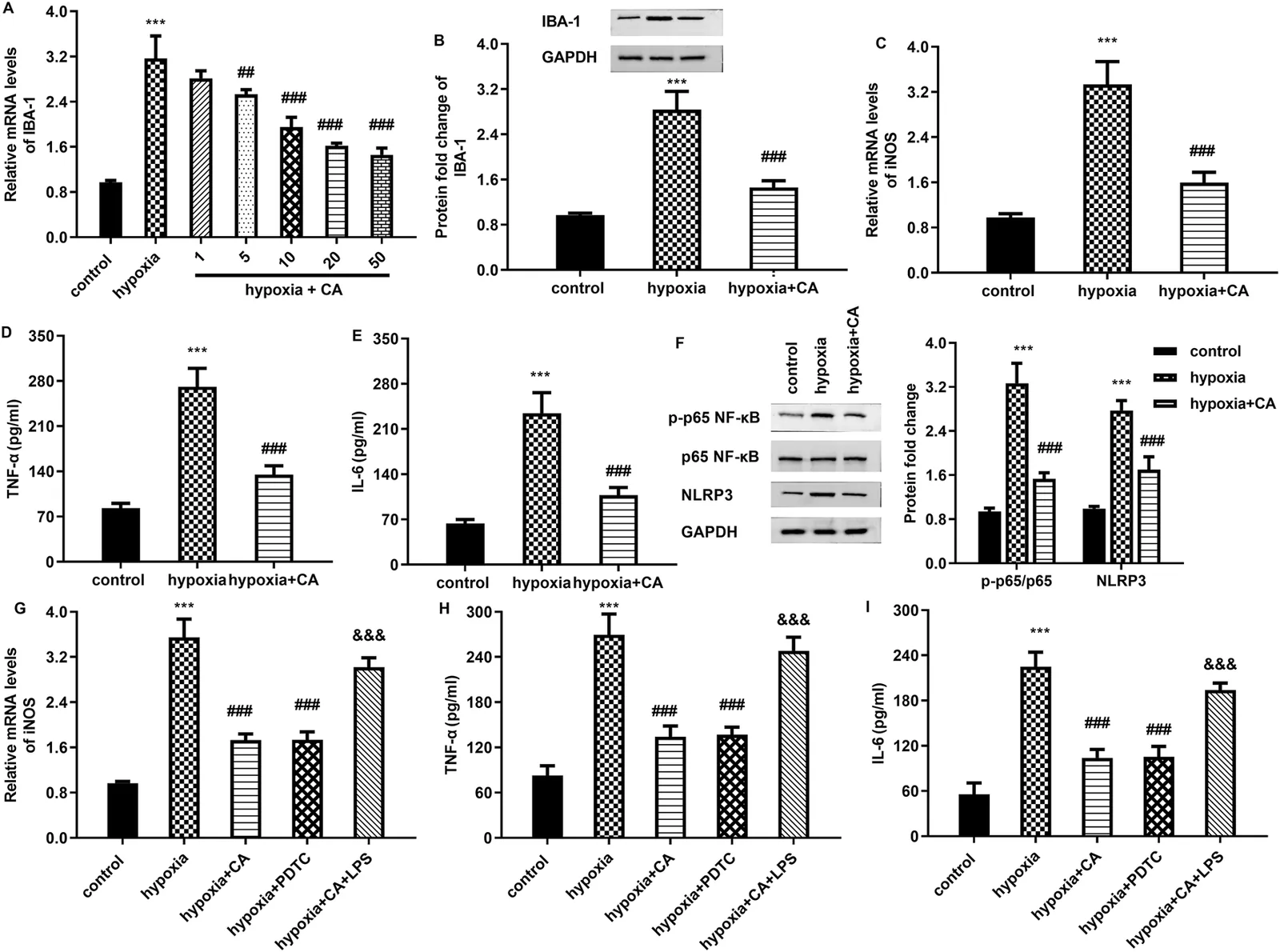
Carnosic acid restrains hypoxia-induced pro-inflammatory microglia activation by inhibiting the NF-κB signaling. **(A)** Microglia were treated with the indicated doses of carnosic acid (1–50 μM) under hypoxia conditions. Then, the mRNA levels of IBA-1 were determined. **(B–E)** Following treatment with 20 μM carnosic acid under hypoxia, the protein expression of IBA-1 **(B)**, transcription levels of iNOS **(C)**, TNF-α **(D)**, and IL-6 **(E)** were detected. **(F)** The effects on the activation of the NF-κB signaling were evaluated by Western assay. **(G–I)** Microglia were treated with carnosic acid, NF-κB signaling activator LPS, and inhibitor PDTC under hypoxia exposure. Then, the mRNA levels of iNOS, TNF-α, and IL-6 were measured. ^***^ *P* < 0.001 vs. control groups. ^##^ *p* < 0.01 and ^###^ *p* < 0.001 vs. hypoxia groups, ^&&&^ *p* < 0.001 vs. hypoxia and CA groups.

### 3.8 CA alleviates pro-inflammatory microglia-mediated injury to astrocytes and endothelial cells

As shown in Figures 8A,B, the increased levels of main aquaporin AQP4 mRNA and protein expression were validated in astrocytes cultured with hypoxia-treated microglial medium (HM), which were reduced when cells were cultured with CA-treated microglia medium. Moreover, after co-culture with hypoxia-activated microglia, the decreased expression of the tight junction-related proteins occludin and ZO-1 was demonstrated in cerebral microvascular endothelial cells, which was reversed after co-culture with CA-treated microglia (Figures 8C,D). The TEER and transcytosis assay revealed damage to tight junction integrity (Figure 8E) and permeability of endothelial cells (Figure 8F) after co-culture with microglia under hypoxic conditions; however, these changes were offset when cells were co-cultured with CA-treated microglia. Thus, CA may attenuate hypoxia-activated microglia-induced damage to astrocytes and endothelial tight junctions.

**FIGURE 8:**
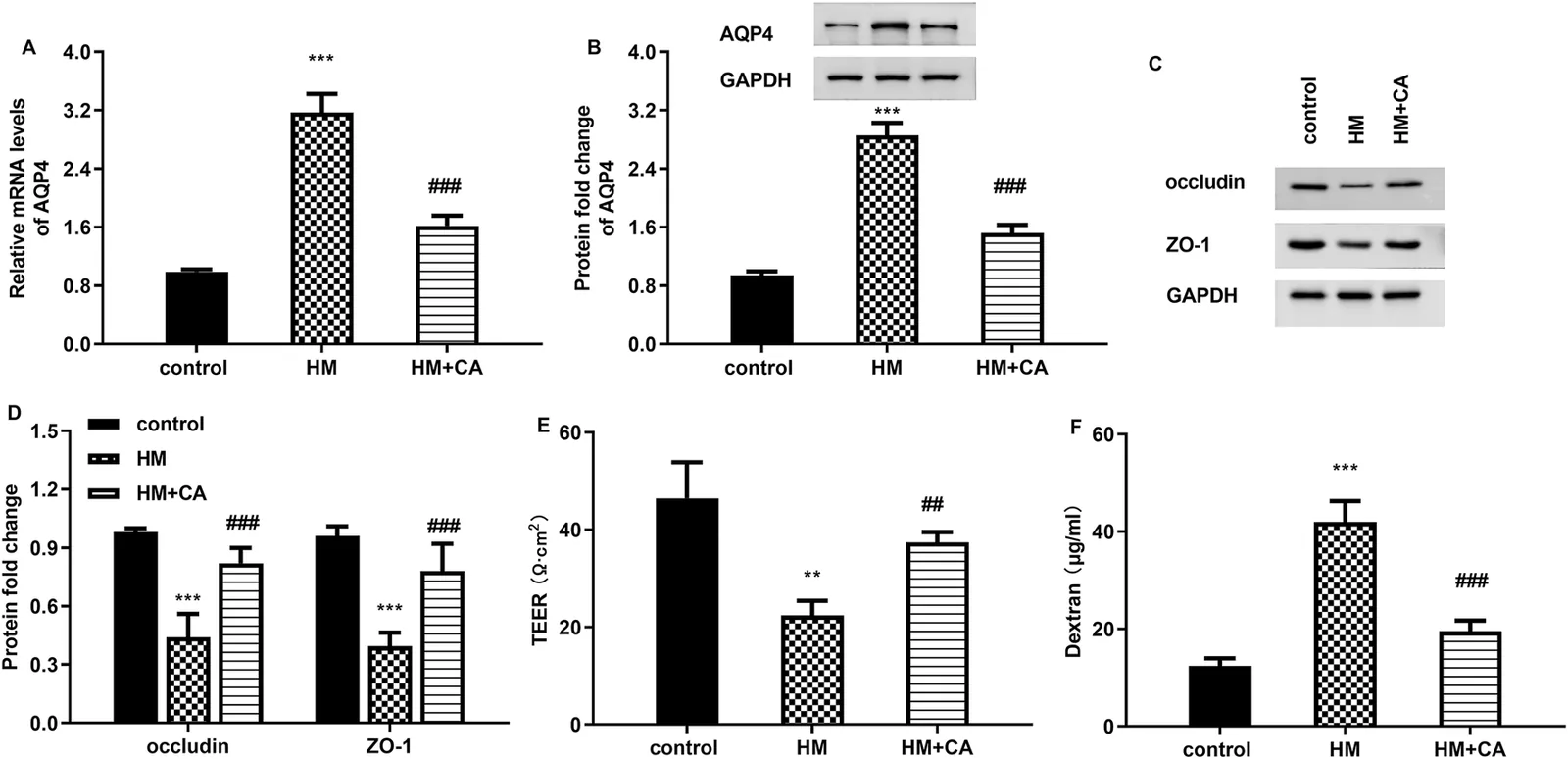
Carnosic acid alleviates pro-inflammatory microglia-mediated injury to astrocytes and endothelial cells. **(A,B)** Rat microglia were cultured with the conditioned medium from hypoxia microglia (HM) and carnosic acid-stimulated microglia under hypoxia conditions. Then, the mRNA **(A)** and protein **(B)** levels of AQP4 were determined in astrocytes. **(C,D)** Rat cerebral microvascular endothelial cells were co-cultured with microglia under carnosic acid and hypoxia conditions. Then, the protein levels of occludin and ZO-1 were analyzed. **(E,F)** TEER **(E)** and transcytosis assays **(F)** were performed to evaluate tight junction integrity and permeability of endothelial cells. ^***^ *p* < 0.001 vs. control groups. ^###^ *p* < 0.001 vs. HM groups.

## 4 Discussion

HACE can develop from the progression of AMS and present with serious clinical symptoms. Accumulating studies have confirmed oxidative stress and neuroinflammation as the pathological contributors to HACE (Pena et al., 2022; Shushanyan et al., 2024). Carnosic acid has been implicated in multiple biological characteristics, including anti-inflammatory and anti-oxidative stress ability (Chrastina et al., 2022; Danisman et al., 2023; Yi-Bin et al., 2022). Up to now, its roles in HACE have remained elusive. This research first substantiated that carnosic acid attenuated HACE-evoked hematological disorders and improved brain edema by attenuating damage to BBB permeability and energetic metabolism disorder. Moreover, carnosic acid alleviated cognitive and motor impairment in HACE rats. CA also reduced HACE-evoked oxidative stress injury and microglia activation-mediated neuroinflammation and inhibited activation of the NF-κB-NLRP3 inflammasome signaling. Thus, these data may reveal the protective efficacy of carnosic acid against HACE, supporting it as a promising agent for the treatment of HACE.

After rapidly ascending to high altitude, healthy people will experience low blood oxygen saturation that will induce increased erythropoietin and red blood cells, leading to polycythemia (Goldfarb-Rumyantzev and Alper, 2014). Moreover, people under a hypobaric, hypoxic environment also experience an increase in hematological parameters (e.g., white blood cells, hematocrit, red blood cell count, and hemoglobin) as a compensatory response to hypoxia. In this study, we first explored the roles of carnosic acid in hematological parameter changes and confirmed that rats under HACE conditions had increased hematological changes, which were consistent with a previous study (Ma et al., 2024; Patir et al., 2012). Previous research revealed similar changes in hematological parameters in young Chinese males under a high-altitude HH environment (Wang K. et al., 2018). Notably, carnosic acid dose-dependently attenuated HH-evoked hematological parameter changes and brought them closer to the normal range.

Next, decreased ATP, Na^+^-K^+^-ATPase, and Ca^2+^-Mg^2+^-ATPase and increased PK levels were observed in HACE rats, indicating a disorder of energy metabolism in HACE. ATP generation exerts a key function in brain energy metabolism. PK is a key enzyme in glycolysis and can facilitate conversion to ATP. Na^+^-K^+^-ATPase and Ca^2+^-Mg^2+^-ATPase can pump Na^+^ and Ca^2+^ out of cells and K^+^ into cells against their concentration gradients by ATP hydrolysis to maintain high intracellular K^+^ and low Ca^2+^ and Na^+^ concentrations (Jeremias et al., 2012). The reduced ATP generation results in energy metabolism disorder and accumulation of intracellular Na^+^ and Ca^2+^, leading to water excess and calcium overload in brain cells and finally to cytotoxic brain edema (Gu et al., 2016; Wang K. et al., 2018). Accumulating evidence confirms energy metabolism disorders in HACE (Gu et al., 2016; Jeremias et al., 2012; Wang G. et al., 2018). Notably, carnosic acid administration alleviated energy metabolism disorder in HACE, supporting it as a neuroprotective mechanism for HACE.

High-altitude environments represent extremely low barometric pressure and oxygen deficiency, leading to accumulation of endogenous ROS. Abundant studies have revealed high oxidative stress status with reduced antioxidative stress levels in HACE (He et al., 2024). The generation of abundant ROS can damage endothelial cells of the BBB and cause extracellular edema, contributing to the progression of HACE (He et al., 2024; Pena et al., 2022; Shushanyan et al., 2024). Here, rats with HH exposure had a high oxidative stress status, as indicated by increased ROS and MDA levels and decreased levels of the antioxidants SOD and GSH. Carnosic acid antagonized high oxidative stress levels in HACE rats. Currently, lowering oxidative stress represents a promising strategy for the treatment of HACE (Gong et al., 2018; He et al., 2024). Of note, carnosic acid possesses cytoprotective effects on chlorpyrifos-induced oxidative damage in brain tissues (AlKahtane et al., 2020). CA can readily cross the BBB and exert neuroprotective efficacy by anti-oxidative effects in experimental models of Parkinson’s disease and Alzheimer’s disease (Iorio et al., 2022). Therefore, carnosic acid may attenuate the progression of HACE by inhibiting oxidative stress levels.

Microglia, the major innate brain-resident immune cells in the central nervous system, can act as the key regulator for neuronal function, neuroinflammation, and behavior in brain disorders (Woodburn et al., 2021). Activated microglia are strongly related to neuroinflammation and can increase vascular permeability, leading to BBB injury and exacerbation of cytotoxic edema in nervous system diseases, including HACE (Geng et al., 2022; Wang et al., 2022). In this study, the increased number of activated microglia and increased pro-inflammatory and decreased anti-inflammatory cytokine levels were validated in HACE rats, indicating the enhanced neuroinflammation in HACE, which is similar to a previous study (Zhou et al., 2017). Here, the increased neuroinflammation was attenuated by carnosic acid in HACE rats. Of note, a previous finding highlighted that carnosic acid could attenuate brain damage by inhibiting inflammation (Liu et al., 2019). CA also alleviates glia-mediated neuroinflammation and improves cognitive function in Alzheimer’s disease (Yi-Bin et al., 2022). Carnosic acid exerts crucial roles in neurodegenerative diseases by regulating oxidative stress and microglia-mediated neuroinflammation (Iorio et al., 2022). Growing evidence supports anti-neuroinflammation as a potential approach against HACE (Geng et al., 2022; Gong et al., 2018). Thus, carnosic acid may alleviate the progression of HACE by suppressing neuroinflammation.

This study highlighted that rats with HACE had higher levels of brain water content, a common marker for brain edema. The blood–brain barrier (BBB) plays important roles in maintaining normal brain function; however, high-altitude HH exposure will compromise the integrity of the BBB and increase its permeability, contributing to cerebral edema. Many experiments have revealed BBB dysfunction in the HACE model, which will evoke vasogenic edema (Jing et al., 2022; Pan et al., 2020). Carnosic acid suppressed BBB permeability, antagonized the decreased expression of BBB-related occludin and ZO-1, and upregulated AQP4 under HACE conditions. Furthermore, *in vitro*, carnosic acid inhibited hypoxia-induced pro-inflammatory microglia activation and activation-induced AQP4, the main aquaporin in astrocytes. Carnosic acid also attenuated hypoxia-microglia-evoked damage to tight junction integrity and the permeability of endothelial cells.

Previous research highlighted that hypoxia-activated microglia can induce astrocyte swelling by inducing AQP4 and disrupting the tight junctions of cerebral microvascular endothelial cells (Wang et al., 2022). Therefore, carnosic acid may regulate BBB permeability by affecting microglia activation-induced astrocyte swelling and the tight junctions of endothelial cells. Of note, a previous study has corroborated carnosic acid as a promising agent to protect brain cells (de Oliveira, 2018). Carnosic acid alleviated brain edema and BBB permeability and improved neurologic function in early brain injury (Teng et al., 2019). Thus, carnosic acid may protect against HACE-induced brain edema and BBB permeability.

This study corroborated that carnosic acid inhibited activation of the NF-κB-NLRP3 inflammasome axis. Inhibiting the NF-κB pathway using PDTC restrained hypoxia-induced pro-inflammatory microglia activation, whereas activating this pathway by LPS reversed this effect, suggesting that carnosic acid could inhibit hypoxia-induced pro-inflammatory microglia activation by suppressing the NF-κB–NLRP3 pathway. NF-κB is a prototypical and classic pathway and can activate downstream signaling to regulate various pathological processes, such as inflammation. For instance, inhibition of the NF-κB–NLRP3 pathway attenuated neuroinflammation and oxidative stress (Che et al., 2024). Similar to Chen et al. (2022) and Geng et al. (2022), the aberrant activation of the NF-κB signaling was observed in the HACE model. Furthermore, inhibiting the NF-κB pathway has been demonstrated to be a promising strategy for high-altitude hypobaric hypoxia-induced brain injury and cerebral edema by alleviating oxidative injury, inflammatory response, and BBB damage (Jiang et al., 2022; Pan et al., 2020). Targeting the NF-κB pathway can attenuate neuroinflammation and BBB disruption and ultimately alleviate the pathological progression of HACE (Chen et al., 2022; Geng et al., 2022; Pan et al., 2020). Thus, these findings suggest that the NF-κB signaling may be involved in carnosic acid-mediated protection against HACE.

Together, the current data first substantiated that carnosic acid alleviated brain edema by inhibiting oxidative stress and microglia activation-mediated neuroinflammation and improved energy metabolism disorder. CA also improved the cognitive and motor dysfunction in the HACE model and inhibited activation of the NF-κB–NLRP3 inflammasome axis. Therefore, this study may support carnosic acid as a promising therapeutic agent for HACE. However, this study only confirmed that carnosic acid inhibited the activation of the NF-κB–NLRP3 inflammasome axis in HACE. Whether this pathway is responsible for carnosic acid-mediated protection in HACE is an open question. Are other pathways involved in the above processes? These questions will be elucidated in our next work.

Several critical questions remain to be addressed in future work. First, although our data confirm an association between carnosic acid and NF-κB–NLRP3 axis-mediated pro-inflammatory microglia activation under hypoxic conditions, this study does not assess critical upstream regulatory molecules (e.g., phosphorylation/degradation of IκBα or IKK activation status) nor does it employ genetic approaches such as gene silencing or overexpression for rescue experiments. Thus, our data may provide limited support for causal relationships within the pathway. Second, the upstream molecular mechanisms by which carnosic acid activates this signaling pathway remain incompletely elucidated. Does carnosic acid directly activate the NF-κB-NLRP3 axis or indirectly regulate this signaling through interaction with other molecules? This mechanism should be explored in future work.

Third, in this study, carnosic acid restored cerebral ATP levels and ATPase activities in HACE rats. However, this study does not distinguish whether this improvement in energy metabolism is a direct effect of the compound on mitochondrial function/glycolytic pathways or merely a secondary consequence of reduced oxidative damage and neuroinflammation. Fourth, our study only analyzed the effects of carnosic acid on pro-inflammatory M1 activation. Emerging evidence confirms that shifting microglial M1/M2 polarization alleviates the progression of HACE (Wang et al., 2023). Does carnosic acid drive microglial polarization toward an anti-inflammatory M2 phenotype? Does carnosic acid attenuate neuroinflammation in the progression of HACE by regulating microglial polarization from a proinflammatory phenotype to an anti-inflammatory M2 phenotype? These questions will be explored in our next plan.

Fifth, as a pharmacodynamic study, no pharmacokinetic data measuring brain tissue or cerebrospinal fluid concentrations of carnosic acid were determined in this HACE model. In addition, this study did not conduct head-to-head comparisons with currently used clinical drugs for HACE treatment (such as dexamethasone and acetazolamide), thereby preventing an assessment of carnosic acid’s relative efficacy and advantages. The absence of a positive drug control group may diminish the clinical translational relevance of this study. The above questions will be explored in the next plan. Future studies will further compare the therapeutic efficacy of CA to current positive drugs (such as dexamethasone and acetazolamide) in animal models or clinical trials to strengthen the clinical translational significance of this study.

In this study, CA dose-dependently ameliorated HH-induced elevation of hematological parameters, and no obvious difference was observed between the 40 mg/kg and 80 mg/kg-treated groups. These findings might be due to target saturation or poor water solubility of the compound. However, which of these reasons is responsible for the above results remains unclear and must be further verified in our future work.

## Data Availability

The raw data supporting the conclusions of this article will be made available by the authors, without undue reservation.
